# *Streptococcus suis* serotype 9 in Italy: genomic insights into high-risk clones with emerging resistance to penicillin

**DOI:** 10.1093/jac/dkad395

**Published:** 2023-12-28

**Authors:** Francesca Romana Massacci, Lucilla Cucco, Marta Panicciá, Andrea Luppi, Elisa Albini, Arianna Peruzzo, Laura Ferroni, Martina Ustulin, Massimiliano Orsini, Chiara Francesca Magistrali

**Affiliations:** Istituto Zooprofilattico Sperimentale dell’Umbria e delle Marche ‘Togo Rosati’, Perugia, Italy; Istituto Zooprofilattico Sperimentale dell’Umbria e delle Marche ‘Togo Rosati’, Perugia, Italy; Istituto Zooprofilattico Sperimentale dell’Umbria e delle Marche ‘Togo Rosati’, Perugia, Italy; Istituto Zooprofilattico Sperimentale della Lombardia e dell’Emilia Romagna, Parma, Italy; Istituto Zooprofilattico Sperimentale dell’Umbria e delle Marche ‘Togo Rosati’, Perugia, Italy; Istituto Zooprofilattico Sperimentale delle Venezie, Padova, Italy; Istituto Zooprofilattico Sperimentale dell’Umbria e delle Marche ‘Togo Rosati’, Perugia, Italy; Istituto Zooprofilattico Sperimentale delle Venezie, Padova, Italy; Istituto Zooprofilattico Sperimentale delle Venezie, Padova, Italy; Istituto Zooprofilattico Sperimentale dell’Umbria e delle Marche ‘Togo Rosati’, Perugia, Italy

## Abstract

**Background:**

*Streptococcus suis* is an important pig pathogen and an emerging zoonotic agent. In a previous study, we described a high proportion of penicillin-resistant serotype 9 *S. suis* (SS9) isolates on pig farms in Italy.

**Objectives:**

We hypothesized that resistance to penicillin emerged in some SS9 lineages characterized by substitutions at the PBPs, contributing to the successful spread of these lineages in the last 20 years.

**Methods:**

Sixty-six SS9 isolates from cases of streptococcosis in pigs were investigated for susceptibility to penicillin, ceftiofur and ampicillin. The isolates were characterized for ST, virulence profile, and antimicrobial resistance genes through WGS. Multiple linear regression models were employed to investigate the associations between STs, year of isolation, substitutions at the PBPs and an increase in MIC values to β-lactams.

**Results:**

MIC values to penicillin increased by 4% each year in the study period. Higher MIC values for penicillin were also positively associated with ST123, ST1540 and ST1953 compared with ST16. The PBP sequences presented a mosaic organization of blocks. Within the same ST, substitutions at the PBPs were generally more frequent in recent isolates. Resistance to penicillin was driven by substitutions at PBP2b, including K479T, D512E and K513E, and PBP2x, including T551S, while reduced susceptibility to ceftiofur and ampicillin were largely dependent on substitutions at PBP2x.

**Conclusions:**

Here, we identify the STs and substitutions at the PBPs responsible for increased resistance of SS9 to penicillin on Italian pig farms. Our data highlight the need for monitoring the evolution of *S. suis* in the coming years.

## Introduction

*Streptococcus suis* is one of the main pathogens in pigs worldwide and an emerging zoonotic agent in humans.^1^*S. suis* infection occurs most commonly in piglets, causing meningitis, septicaemia, polyserositis, arthritis and endocarditis. Transmission to humans occurs from occupational contact or the consumption of undercooked pork.^2^ So far, 29 serotypes of *S. suis* are recognized, based on the capsule polysaccharides.^1^ Among them, serotype 2 is the most frequently isolated worldwide in pigs and humans.^1^ However, the prevalence of serotype 9 has increased in China and in several European countries, where it has become the dominant serotype recovered from diseased pigs.^1,3–5^

In a previous study,^6^ we analysed the serotype distribution, the clonal composition and the antibiotic susceptibility of *S. suis* isolates causing streptococcosis in pigs in Italy during 2017–19. In line with what has been reported in other European countries, we described a shift of the dominant serotype from serotype 2 to serotype 9.^1,3–5^ Surprisingly, resistance to penicillin was recorded in one out of five isolates in the collection. Such resistance was strictly associated with SS9, with more than half of SS9 isolates being clinically resistant to penicillin.

β-Lactams represent the first-line antibiotic therapy against *S. suis* infections.^7,8^ The targets of this antibiotic class are the PBPs, which are involved in the biosynthesis of the bacterial cell wall. β-Lactams covalently bind to the active domains of the PBPs, transpeptidase or carboxypeptidase, which harbour the active motifs SXXK, (S/Y)XN and (K/H)(D/T)G. Such binding prevents the peptide crosslinking of the glycan strains, a crucial step in the synthesis of peptidoglycan.^9,10^ Resistance has been extensively studied in *Streptococcus pneumoniae*, in which the main resistance mechanisms are mutations in the active sites of the PBP sequences, which reduce β-lactam binding affinity.^9,11–15^*S. suis* is traditionally considered susceptible to β-lactams. However, strains resistant to this antibiotic class are increasingly reported worldwide. In Thailand, approximately 8% of strains of human origin showed a reduced susceptibility to penicillin. In China, the proportion of *S. suis* resistant to β-lactams on pig farms increased from 11.6% in 2013 to 19.7% in 2017.^16^ Regarding Europe, resistance to β-lactams was low in isolates from Denmark, Sweden and France, ranging from 1% to 5%.^17–19^ By contrast, more than one-fifth of isolates from diseased pigs in Spain and in Italy were found to be resistant to penicillin.^6,20^ Similarly to what was reported for other members of the genus, decreased susceptibility to β-lactams was linked to the presence of variant PBPs including 1a, 2b, 2a and 2x.^5,21–23^

The hypothesis behind this study is that resistance to penicillin emerged in some SS9 lineages and contributed to their successful spread, as a possible consequence of the use of β-lactams on Italian pig farms. To confirm this hypothesis, we performed a retrospective analysis of SS9 isolates collected from diseased pigs in Italy between 2002 and 2021. We tested the isolates for susceptibility to penicillin and other β-lactams, such as ceftiofur and ampicillin. We found that reduced susceptibility to β-lactams was associated with the ST and the year of isolation. Furthermore, amino acid alteration patterns at the PBPs were predictive of increased MICs of β-lactams.

## Materials and methods

### S. suis isolates

We investigated isolates collected from pigs with clinical *S. suis* infection on pig farms in nine regions of northern/central Italy during 2002–21 belonging to serotype 9. The isolates were collected by four diagnostic laboratories. A proportion of these isolates (*n* = 25) were analysed in our previous study.^6^ To avoid redundancy, we included only one isolate per year and farm. A total of 66 SS9 isolates were selected from diseased piglets as shown in Table S1 (available as Supplementary data at *JAC* Online). The samples were cultured as already described.^6^

### Antimicrobial susceptibility testing

We assessed MICs by using an MIC panel (BOP06F, Sensititre; Trek Diagnostic Systems Inc.) according to the manufacturer’s instructions. *S. pneumoniae* ATCC 49619 was used as a quality control strain. MIC values were interpreted using the breakpoints recommended by CLSI for *S. suis,* when available.^24^ The interpretative criteria for trimethoprim/sulfamethoxazole and clindamycin were those recommended for human *S. pneumoniae*.^25^

### WGS

In order to investigate ST, virulence profile and antimicrobial resistance genes, 66 SS9 isolates were whole-genome sequenced. Genomic DNA of the *S. suis* cultures was extracted using QIAamp DNA Mini Kit (QIAGEN Inc., Hilden, Germany) following the manufacturer’s protocol for Gram-positive bacteria. Each sample was then quantified with the Qubit fluorometer (Qubit™ DNA HS Assay, Thermo Fisher Scientific Inc.). Libraries were prepared using the Nextera XT Library Prep kit (Illumina Inc., San Diego, CA, USA) and then sequenced on an Illumina NextSeq platform to generate 150 bp paired-end reads.

### Bioinformatic analysis

Illumina reads were trimmed and checked for quality using Fastp v0.19.5^26^ with default parameters, assembled using SPAdes genome assembler v3.11.1,^27^ checked for quality assessment of draft genome sequences with QUAST v5.0.2^28^ and annotated using Prokka v1.14.6.^29^ The resulting general feature formats (GFFs) produced by Prokka were analysed with Roary v3.11.3^30^ to obtain a core-genome alignment used to create a maximum-likelihood phylogenetic tree (FastTree 2.1.11).^31^ Manual annotation of the tree was performed in iTOL (v. 6, https://itol.embl.de/). In order to compare our isolates with the ones available in literature, we downloaded the genomes referring to SS9 in the Sequence Read Archive (SRA) database. The genomes were included in further analyses. Highly fragmented assemblies and those with possible contamination (greater than 200 contigs) were removed. The final collection contained 40 genomes. The dataset included 13 strains from Canada, 18 from the UK, 5 from Spain, 2 from China, 1 from Denmark and 1 from Brazil. Those selected isolates were previously described by Zheng *et al.*^1^ and Hadjirin *et al.*^23^*In silico* MLST analysis was performed, submitting sequences to the *S. suis* MLST database to obtain allele number and ST. The new allele sequences or STs were submitted to the database curator.

Virulence genes were searched using BLASTN v2.13.0+,^32^ creating a database of 119 previously described genes^2,33^ and considering only genes with ≥95% coverage and ≥99% identity.^34^

Antibiotic resistance genes were analysed with ABRicate (https://github.com/tseemann/abricate). The cut-offs used to determine antimicrobial resistance gene presence were the same as for virulence profiling (≥95% coverage and ≥99% identity). Our investigation of β-lactam resistance focused on PBP mutations within PBP1a, PBP1b, PBP2a, PBP2b and PBP2x genes. PBP sequences of the 66 isolates were manually aligned running MUSCLE online (https://www.ebi.ac.uk/Tools/msa/muscle/) and using *S. suis* BM407 (GenBank GCA_000026745.1) as reference. We submitted the raw sequencing data to the NCBI SRA [BioProject ID PRJNA717238 (accession numbers SAMN18490763, SAMN18490790, SAMN18490771 and SAMN18490773)—BioProject ID PRJNA962302 (accession numbers SAMN34394129–SAMN34394143)].

### Statistical analysis

A heatmap was produced to represent the distribution of virulence genes across the isolates. Differences among STs in the total number of virulence genes detected per isolate were then evaluated using the Kruskal–Wallis and *post hoc* Dunn test, after checking the normality of data with the Shapiro–Wilk test. Multiple linear regression models were employed to investigate factors associated with an increase in MICs of penicillin, ceftiofur and ampicillin, respectively. The response MIC was log-transformed and separate models were built using penicillin, ceftiofur and ampicillin. A first set of analyses aimed to test possible effects of the ST and the year of isolation. A second set was then performed to explore potential associations with the substitutions at the PBPs. STs represented by only one isolate were grouped as ‘other’, and ST16 was employed as baseline. ST16 was chosen because of its importance as a zoonotic pathogen and the absence of significant changes in MIC values throughout the study period. Before testing the effect on susceptibility to β-lactams, substitutions at the PBPs were grouped according to their distribution in the heatmap and then confirmed as belonging to the same group (PBP pattern) if they showed a tetrachoric index of ≥0.8. A variable was removed if a single category accounted for over 85% of isolates (>56). An iterative forward stepwise approach was employed to select variables that improved the model fit and build models retaining only statistically significant factors. Statistical analyses were performed using R (v 4.1.3), setting the significance level at α = 0.05.

## Results and discussion

The characteristics of the 66 *S. suis* isolates are shown in Table S1. Thirteen different STs were identified, with ST123 and ST16 being the most prevalent, accounting for 53% (*n* = 35) and 13.6% (*n* = 9) of our collection, respectively. Five new STs were identified as ST1953–ST1957 (ID3308-ID3314; https://pubmlst.org/organisms/streptococcus-suis).

When compared with other SS9 genomes available in public repositories, the 42 Italian isolates belonging to ST123, ST1953 and ST94 grouped in the same cluster with 5 Spanish strains belonging to ST123 and ST125 (Figure S1). One Canadian strain (ST54) and an Italian ST1546 were grouped in a second cluster. SS9 isolates from Italy belonging to ST16 and one ST136 were in the third cluster, while ST1540 isolates were placed within the fourth cluster. The fifth cluster consisted of 31 isolates from four different countries (the UK, Brazil, Canada and China). Thus, we confirmed the genetic heterogeneity of SS9 strains already reported in the literature.^1^ The presence of ST123 seems to be confined to Spain and Italy, in agreement with another report.^1^

To better characterize these emerging STs, we investigated the presence of putative virulence factors and antibiotic resistance in our sample collection. We retrieved 86 putative virulence genes and included them in a heat map (Figure S2). Kruskal–Wallis test showed an overall significant difference among different STs in the total number of putative virulence genes detected in each isolate (*P* < 0.001). In particular, ST123 differed from ST16, while ST1540 differed from ST16 and ST94 (*P* < 0.05 by Dunn test). A block of 18 putative virulence genes was characteristic of ST123 and ST94 isolates. This block included genes encoding molecules related to adhesion (*srtF*), putative virulence genes involved in the regulation of metabolic pathways (*lspA*, *sodA*, *adcR*, *scrB, ppc*, *troA*, *nox*, *purD*, *msmK*, *gloA*, *rgg* and *lysM*). Other clusters of putative virulence genes were evident regarding ST16 and ST1540.

**Figure 1.**
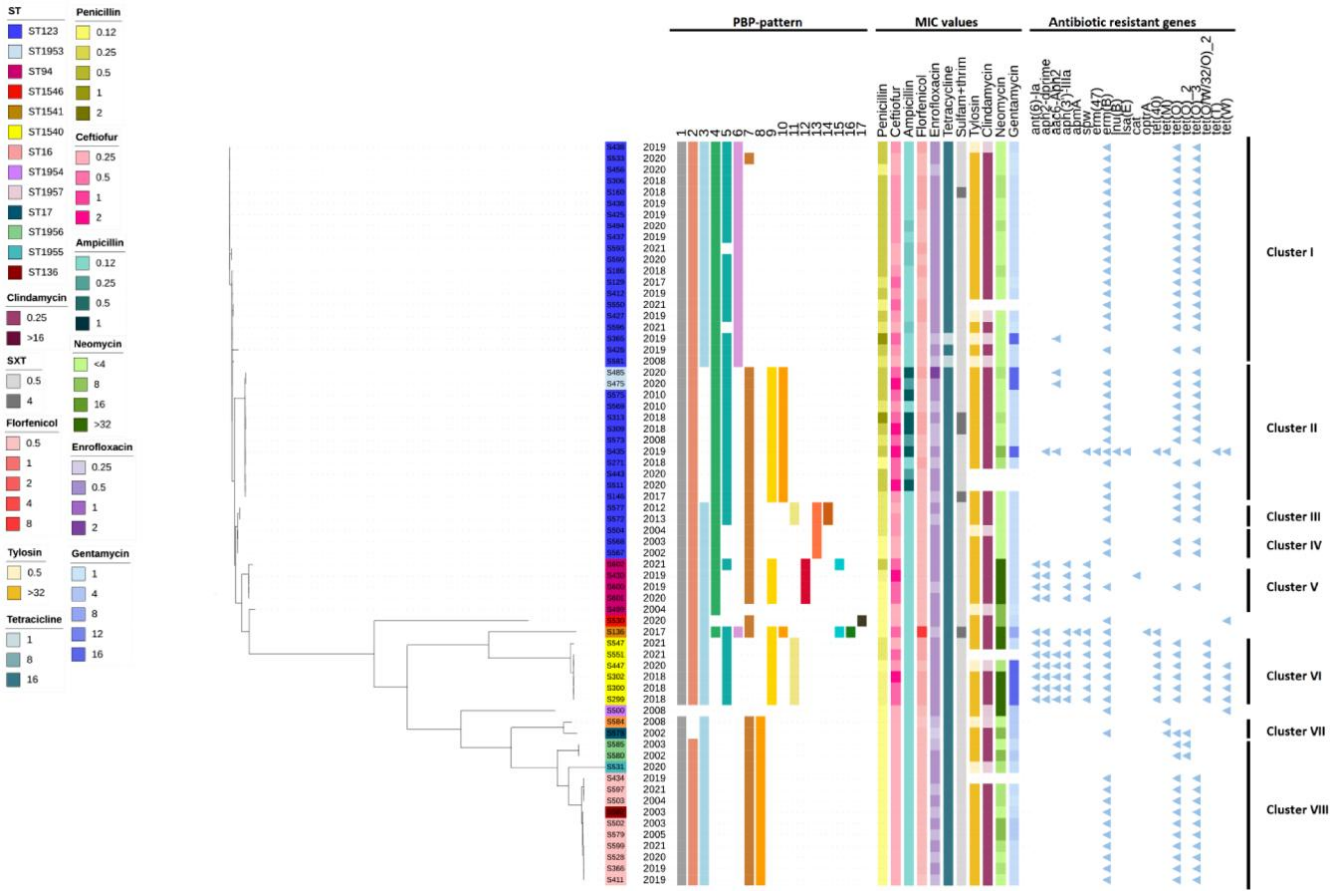
Maximum-likelihood phylogenetic tree containing 66 *S. suis* serotype 9 isolates from Italy. The tree was inferred by using the iTOL interactive user interface (https://itol.embl.de). The STs, the PBP patterns, MIC values and antibiotic resistance genes of each isolate are shown.

The distribution of SS9 isolates according to antibiotic MIC values is shown in Table 1. Consistently with what was already reported,^6^ a high number of isolates were resistant to clindamycin 52/62 (85.2%) and all isolates were resistant to tetracycline 66/66 (100%). The most common (67.6% of isolates) phenotypic profile was resistance to macrolides and tetracyclines, generally associated with the presence of *tet*(O) and *erm*(B). Isolates belonging to ST16 and most of the ST123 isolates showed the *tet*(O)*/erm*(B) profile. By contrast, determinants for resistance to aminoglycosides, including *ant(6)-Ia* and *spw*, were most concentrated in isolates belonging either to ST94 or ST1540 (Figure 1).

**Table 1.**
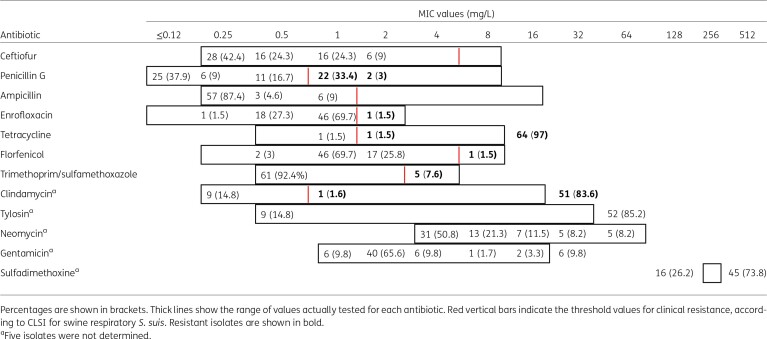
Distribution of MIC values for the 66 *S. suis* isolates

It is worth noting that high MICs of penicillin (≥1 mg/L) were recorded in approximately one-third of our collection (24/66), which were consequently classified as resistant to this antibiotic according to the CLSI breakpoints.^24^

**Figure 2.**
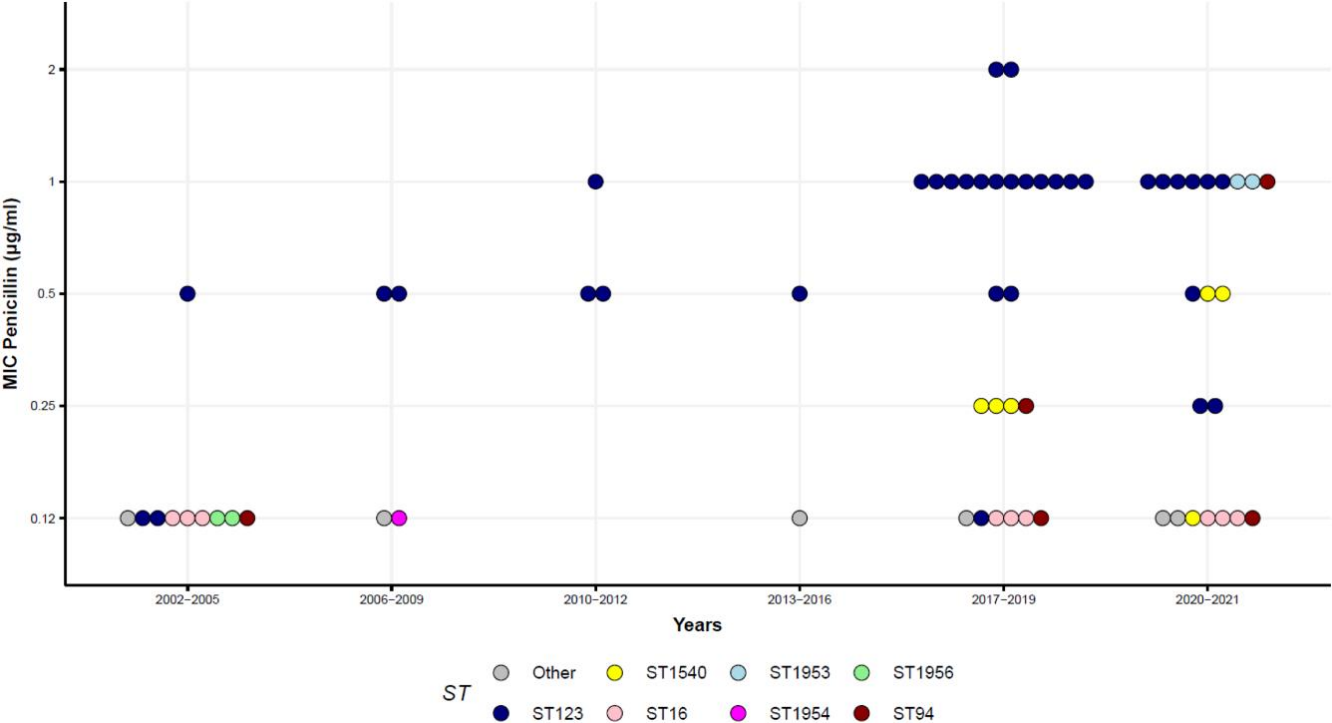
Distribution of the MIC values among the years of isolation. Dots are coloured according to the STs.

Multiple regression analysis showed a positive association between MIC values (log) for penicillin and year of isolation and belonging to ST123 (*n* = 35), ST1540 (*n* = 6) and ST1953 (*n* = 2) compared with ST16 (*n* = 9) (adjusted R^2^ = 0.661) (Figure 2 and Table 2). The model revealed an annual increase in the MIC value for penicillin of approximately 4%, demonstrating that the susceptibility to penicillin decreased significantly from 2004 to 2021 in SS9 Italian isolates. This temporal trend is confirmed by the observation that all penicillin-resistant isolates were isolated after 2017. In addition, resistance to penicillin was not homogeneously distributed in SS9, but linked to some STs. ST123 was associated with MICs of penicillin 5.2-fold higher than those observed in ST16, 1.9 times for ST1540, and 6.7 times for ST1953. Regarding to the other two β-lactams, all isolates with MIC values higher than 2 mg/L (ceftiofur) or 1 mg/L (ampicillin) were observed only from 2018 onwards. However, the association between time, STs and increased MICs of ceftiofur or ampicillin was not confirmed by statistical analysis, probably because of the low number of isolates with reduced susceptibility to ceftiofur and ampicillin. The progressive increase in penicillin resistance over the observation period and its association with some STs are in accordance with what was observed for *S. pneumoniae*, where the spread of successful clones was the main driver for the worldwide decline of susceptibility to β-lactams.^9^

**Table 2.** Effect of year of isolation and ST on the MICs of penicillin log-transformed

Outcome variable: MIC value to penicillin log transformed			Adjusted R^2^ = 0.661	
Factor	Level	Coefficient	95% CI	*P*
Year of isolation	—	0.018	0.007–0.029	0.002
ST	ST16 (baseline)			
	ST123	0.718	0.534–0.903	<0.001
	ST1540	0.281	0.017–0.544	0.037
	ST1953	0.824	0.436–1.212	<0.001
	ST1956	0.215	−0.190–0.621	0.292
	ST94	0.211	−0.063–0.486	0.128
	other	0.035	−0.213–0.283	0.776

We observed that mutations to PBP sequences were associated with amino acid alteration patterns, which were frequent in penicillin-resistant isolates (Table 3, Table S2, Table S3 and Figure S3). This observation is in line with the well-known mosaic structure of PBPs, where sequences of PBPs in isolates resistant to β-lactams show a high number of substitutions compared with susceptible isolates.^9^ Along with substitutions with an effect on the affinity for β-lactams, the patterns probably include substitutions without a specific function and substitutions that restore the functionality of the protein, alleviating their fitness cost.^35,36^ The presence of large blocks of substitutions prevented us from identifying those directly responsible for resistance. This is a common limit of observational studies on PBPs and β-lactam resistance.^9^

**Table 3.** Alteration patterns of the PBPs associated with increased MICs of β-lactams described in the present study

Substitution	ST and number of isolates in brackets	PBP alteration pattern	Effect on MIC values	References
PBP2X_S450T	ST123 (35), ST1541 (1), ST1952 (2), ST94 (5)	4	Increase of a factor of 1.9 and 1.6 in the MICs of penicillin and ceftiofur, respectively	^21,23^
PBP2X_T551S				^21–23^
PBP2X_I568T				^21,23^
PBP2B_N356E	ST123 (32), ST1540 (6), ST1541 (1), ST1952 (2), ST94 (1)	5	Increase of a factor of 3.4 in the MICs of penicillin	^21,23^
PBP2B_S374A				^21,23^
PBP2B_S375G				^21,23^
PBP2B_T376S				^21,23^
PBP2B_Y432W				^21,23^
PBP2B_G433DEL				^21,23^
PBP2B_I452A/V				^21,23^
PBP2B_K479T				^21–23^
PBP2B_D512E				^21–23^
PBP2B_K513E				^21–23^
PBP2B_T515S				^21–23^
PBP2B_T625R				^21,23^
PBP2B_K674N				^21,23^
PBP2X_A627S/T				^21^
PBP2X_V257I	ST123 (10), ST1540 (6), ST1541 (1), ST1952 (2), ST94 (4)	9	Increase of a factor of 2.9 in the MICs of ceftiofur	
PBP2X_N284T				^21,23^
PBP2X_A380E/D				
PBP2X_Y382K/M/Q				^21,23^
PBP2X_M437L				^23,22^
PBP2X_S445T				^21–23^
PBP2X_T491S				^23^
PBP2X_V494L/I				^21,23^
PBP2X_Y525F				^21–23^
PBP2X_D541E				^21,23^
PBP2X_V547M				^21^
PBP2B_D587E				^21,23^
PBP2X_N279T	ST123 (10), ST1541 (1), ST1952 (2)	10	Increase of a factor of 3.5 in the MICs of ampicillin	
PBP2X_R288K				^21,22^
PBP2X_V295I				
PBP2X_Q321A				^21,22^
PBP2X_L324M				
PBP2X_L325I				
PBP2X_V346L				
PBP2X_N364S				
PBP2X_V367T				
PBP2X_L378V				
PBP2X_S383E				
PBP2X_Y389F				^21,22^
PBP2X_M401V				
PBP2X_Q405E				^22^
PBP2X_Q407E				^21,22^
PBP2X_F422Y				^21,22^
PBP2X_T467S				^23,22^
PBP2X_D511A				
PBP2X_R514G				^22^
PBP2X_N569K				^22^
PBP2X_N595S				^21–23^
PBP2X_R600N/D				^22^
PBP2X_D601T/S				^21^
PBP2B_T507I				^21^

Only the substitutions located in the transpeptidase domains of the PBPs are shown.

To overcome this limitation, we analysed the effect of the alteration patterns on reduced susceptibility to β-lactams. The substitutions were grouped in 17 PBP patterns, 1 to 17. These PBP patterns included a variable number of substitutions, ranging from 2 to 67, occurring in just one or more than one PBP sequence. Only six substitutions were not included in any PBP pattern (Table S2).

Penicillin resistance was associated with PBP patterns 4 and 5 (adjusted R^2^ = 0.685), which produced, according to the model (Table S4a), an increase of a factor of MIC of ca. 1.9 and 3.4, respectively. The substitutions included in the PBP patterns associated with increased MICs of β-lactams are described in Table 3. Briefly, PBP pattern 4 included substitutions at PBP2x: among them, T551S and I568T occurred at two active motifs of PBP2x, KSGT and YIN, respectively. PBP2x_T551S was reported to be associated with an increased penicillin MIC.^23^ Conversely, PBP pattern 5 was composed almost exclusively of substitutions at the PBP2b sequence. Among them, substitutions K479T, D512E, K513E and T515S were reported to be associated with an increased penicillin MIC,^23^ while substitution T625R was associated with β-lactam resistance.^22^ Also in PBP pattern 5, PBP2x_A627S/T is located at the C terminal of the protein (Table S2).^9^

A reduced susceptibility to ceftiofur was associated with the presence of PBP patterns 4 and 9 (adjusted R^2^ = 0.650); according to the model (Table S4b), this influence was quantifiable as an increase of a factor of 1.6 and 2.9 in the MIC values, respectively. PBP pattern 9 includes several substitutions to the PBP2x sequence and one substitution to the PBP2b sequence, D587E. Substitutions D541E and V547M occurred in proximity to the active motif KSG of PBP2x. M437L, S445T and Y525F at the PBP2x sequence were associated with high MIC values for ceftiofur^23^ and the last one was also reported as being associated with high MICs of β-lactams (Table 3, Table S2).^22^

Finally, the presence of PBP pattern 10 was associated with reduced susceptibility to ampicillin (adjusted R^2^ = 0.554), with an increase of a factor of approximately 3.5 in the MIC values. PBP pattern 10 was the largest in our study, with substitutions occurring at the PBP2x (*n* = 55) and of PBP2b (*n* = 4) sequences. Substitutions N595S, N569K and T467S at the PBP2x sequence are associated with high MICs of penicillin and ceftiofur,^23^ while substitution PBP2x_Q405E is associated with development of β-lactam resistance (Table 3, Table S2).^22^

Here, we confirmed that development of β-lactam resistance in *S. suis* largely depends on substitutions of the PBP sequences. Despite different areas of origin, serotypes and STs, other investigators have confirmed the majority of the substitutions observed in our study.^21–23^ As previously described for *S. pneumoniae*, different substitutions at the PBP sequences are responsible for penicillin, aminopenicillin and cephalosporin resistance.^14^ The reduced susceptibility to penicillin depends on different substitutions occurring at PBP2b and PBP2x. Conversely, the susceptibility to ceftiofur and ampicillin are mainly affected by substitutions at the PBP2x sequence. Most substitutions associated with reduced susceptibility to β-lactams in our study were present in the genomes of Spanish isolates belonging to ST123 and ST125 (Table S5). Even though data on the antibiotic susceptibility were not available for these strains, the presence of these substitutions suggests that the spread of this high-risk *S. suis* lineage characterized by a reduced susceptibility to β-lactams is not restricted to Italy. Mutations in PBP sequences can be acquired from commensal species by transformation, as already hypothesized for *S. pneumoniae*. In *S. pneumoniae*, the origin of sequences encoding PBPs with a low affinity for β-lactams has been traced back to interspecies recombination with commensal species belonging to the same genus, *Streptococcus mitis* or *Streptococcus oralis*.^9,10,14^ The presence of large blocks of mutations in our isolates suggests a similar mechanism might have occurred in *S. suis*.

The phylogenetic tree confirmed the presence of clusters characterized by a different susceptibility to β-lactams within the collection (Figure 1).

ST16 isolates all grouped together, and showed very low MICs (≤0.25 mg/L) of all the tested β-lactams. Despite being isolated from 2003 to 2021, ST16 isolates showed a low number of substitutions at the PBPs, and none of them belonged to groups associated with increased MIC values to β-lactams. Conversely, resistance to β-lactams was common in ST123 isolates, but with different patterns, depending on the year of isolation and the cluster they belonged to. Cluster I grouped ST123 isolates that were collected from 2018 to 2021 and harboured substitutions belonging to PBP pattern 4 and 5, but not to PBP pattern 9, or 10. These isolates generally showed a moderate increase of MICs of penicillin and ceftiofur. Cluster II was composed of isolates from 2008 to 2020, belonging to ST123 or ST1953, an ST that differed from ST123 by a single allele. This cluster harboured the highest number of substitutions, including those of PBP patterns 4, 5, 9 and 10. These isolates showed moderate (0.5 mg/L) or high (≥1 mg/L) MICs of one or more of the tested β-lactams and particularly to ceftiofur. All the strains showing ampicillin MICs above 0.5 mg/L belonged to cluster II. Finally, the oldest ST123 isolates in our collection, harbouring PBP pattern 4, but not PBP patterns 5, 9 or 10, grouped in cluster IV and showed low MICs of penicillin and ceftiofur (≤0.5 mg/L). These data reinforce the hypothesis of evolution of ST123 to survive the selective pressure generated by the use of β-lactams.

The same trend of an increasing number of substitutions at the PBP sequences over the years was observed in ST94. S499 was isolated in 2004 and harboured substitutions belonging to PBP pattern 4, but not to PBP pattern 5, 9 or 10. S499 showed low MIC values to all the tested β-lactams. ST94 isolates from 2019–20 belonged to cluster V and were characterized by high MICs (1–2 mg/L) of ceftiofur, while MICs of penicillin remained low (0.12–0.25 mg/L). Along with substitutions of PBP pattern 4, these isolates harboured substitutions of PBP pattern 9, but not PBP pattern 5 or 10. Finally, S602, isolated in 2021, was the only ST94 isolate harbouring PBP patterns 4, 5 and 9 and it was resistant to penicillin.

Notably, the only isolate with unmodified PBP sequences in our collection, S500, did not group with other isolates and it was characterized by very low MICs of all the tested β-lactams. S500 originated from a wild boar and therefore, differently from isolates from farmed pigs, did not undergo the selective pressure generated by antibiotic use.

In Italy, antibiotic consumption in livestock has declined by 53% in the last 10 years, according to ESVAC.^37^ However, among EU countries, Italian animal agriculture remains stubbornly high in antibiotic use, and the class of penicillins ranks first among the antibiotic classes used on Italian farms.^37^ Penicillins are suggested as the first-choice antibiotics to treat post-weaning piglets affected by streptococcosis, with aminopenicillins classified as the second choice.^7^ Until 2021, cephalosporins were parenterally administered to piglets to treat severe cases of streptococcosis.^38^ Our data suggest that clones of SS9 with reduced susceptibility to β-lactams have been selected and spread on Italian pig farms over time under antimicrobial selective pressure, representing a threat to animal and human health. We suggest that diagnostic laboratories adopt quantitative methods to assess antibiotic susceptibility in *S. suis*, as some strains with PBP variants may escape scrutiny due to values below the clinical cut-off. In addition, since reduced susceptibility may be observed only in some molecules of this class, penicillin, an aminopenicillin and a third-generation cephalosporin should always be included in the test panel. Here, we identify the amino acid alteration patterns and clones that appear to be reliably responsible for increased resistance of *S. suis* to penicillin on pig farms. Our study reinforces the need for monitoring the evolution of non-susceptible *S. suis* lineages in the coming years.

## Supplementary Material

dkad395_Supplementary_Data

## Supplementary data

Figures S1 to S3 and Tables S1 to S5 are available as Supplementary data at *JAC* Online.
