# Supplementary material for: Streptococcus suis serotype 9 in Italy: genomic insights into high-risk clones with emerging resistance to penicillin
Source: J Antimicrob Chemother. 2023 Dec 28;79(2):403–11. doi: 10.1093/jac/dkad395 (PMC10832592; doi:10.1093/jac/dkad395)

**Figure S1.** Maximum likelihood phylogenetic tree containing 106 *Streptococcus suis* serotype 9 isolates from different countries. The tree was inferred by using the iTOL interactive user interface (<https://itol.embl.de>). The country of origin and the ST of each isolate are shown.


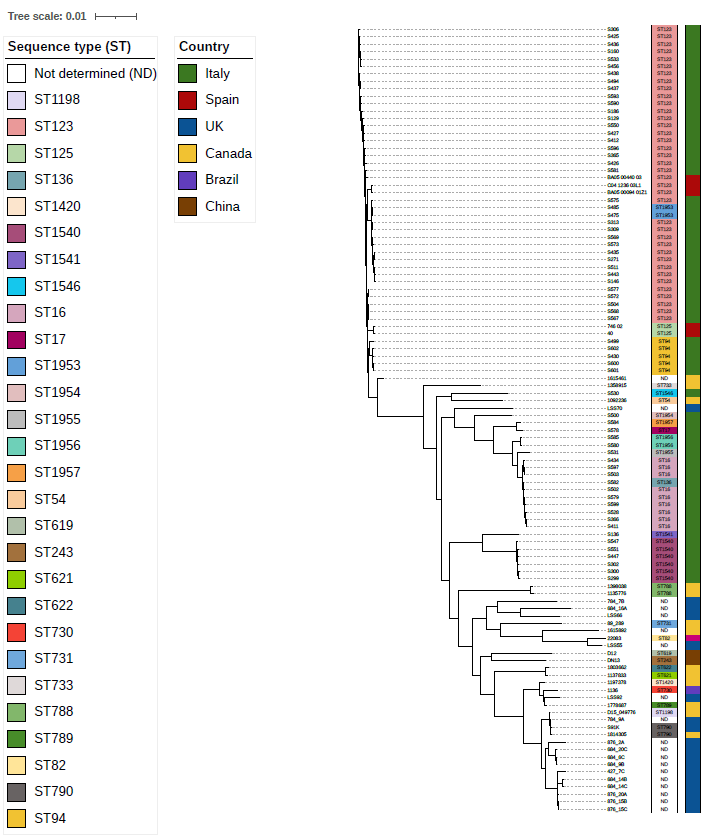


**Figure S2.** Heatmap showing the distribution of putative virulence genes in the 66 *Streptococcus suis* isolates. In light blue the present genes and in white absent genes are represented. The STs for each isolate are also reported.


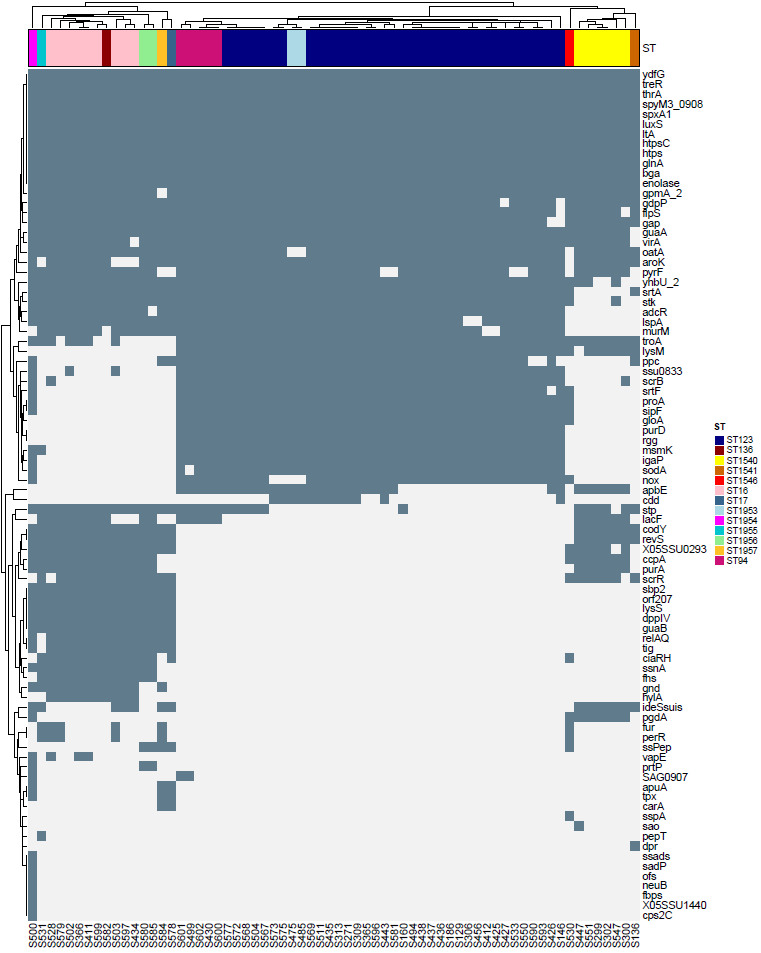


**Figure S3** Substitutions at the transpeptidase domain of PBP1A, PBP2B and PBP2X.


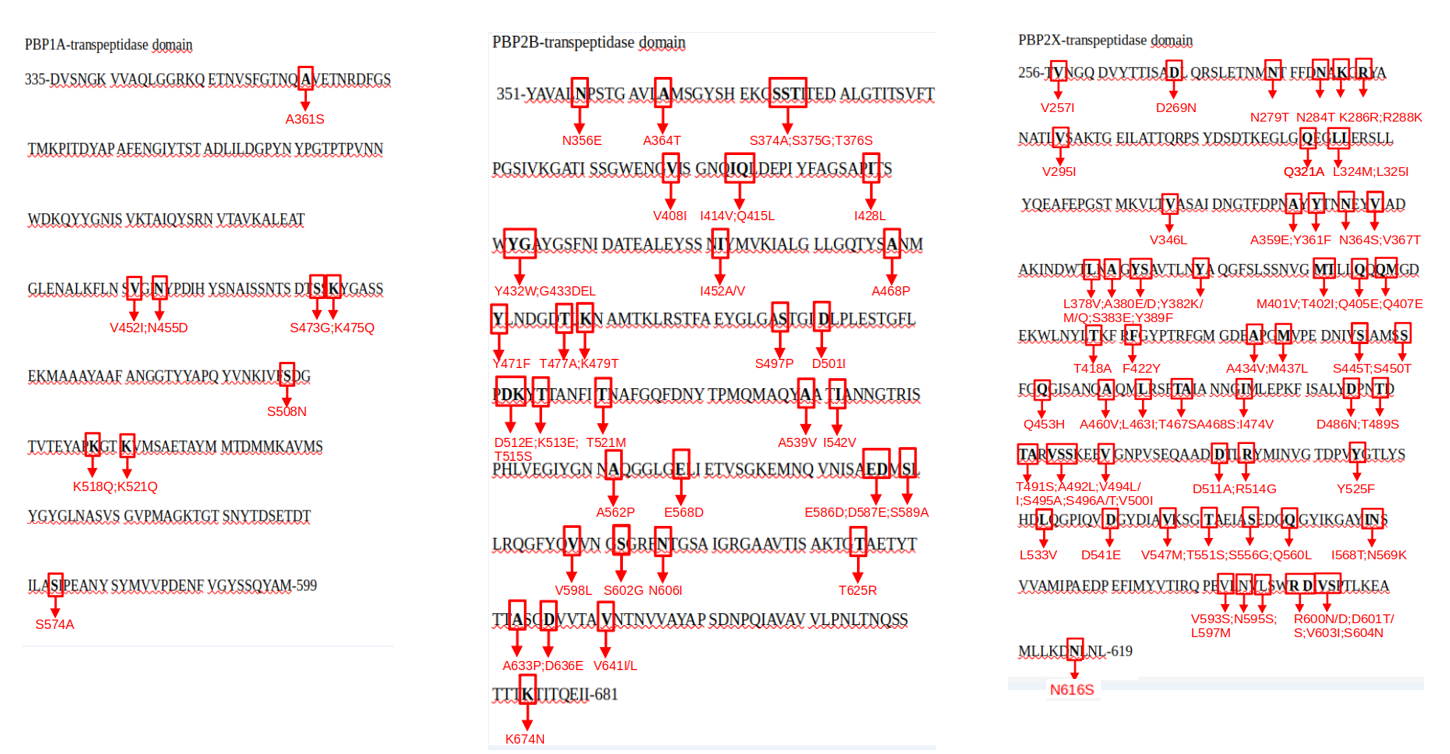

Supplement: dkad395_Supplementary_Data [file dkad395_supplementary_data.zip › Figure_S1_S2_S3_CLEAN.docx]
