# Supplementary material for: Streptococcus suis serotype 9 in Italy: genomic insights into high-risk clones with emerging resistance to penicillin
Source: J Antimicrob Chemother. 2023 Dec 28;79(2):403–11. doi: 10.1093/jac/dkad395 (PMC10832592; doi:10.1093/jac/dkad395)
Supplement: dkad395_Supplementary_Data [file dkad395_supplementary_data.zip › Table_S3-S4_r1_CLEAN.docx]

**Table S3.** Amino acid substitutions in PBP1A, PBP1B, PBP2A, PBP2X and PBP2X in the 24 penicillin resistant *Streptococcus suis* (MIC ≥1 mg/L)

| **Strain** | **ST** | **Penicillin** | **Ceftiofur** | **Ampicillin** | **PBP1A substitutions** | **PBP1B substitutions** | **PBP2A substitutions** | **PBP2B**  **substitutions** | **PBP2X**  **substitutions** |
| --- | --- | --- | --- | --- | --- | --- | --- | --- | --- |
| S160 | ST123 | 1 | 0,5 | 0,12 | PBP1A_T685A, PBP1A_A710S, PBP1A_G734D | PBP1B_E576K, PBP1B_T578A, PBP1B_A765P, PBP1B_S782A, PBP1B_N789T | PBP2A_H27R, PBP2A_I34V, PBP2A_I121V, PBP2A_N233D, PBP2A_A561S, PBP2A_S660T | PBP2B_P92S, PBP2B_D115N, PBP2B_K143E, PBP2B_N179S, PBP2B_S206A/V, PBP2B_I300V, PBP2B_R332K, PBP2B_N356E, PBP2B_S374A, PBP2B_S375G, PBP2B_T376S, PBP2B_I414V, PBP2B_Y432W, PBP2B_G433DEL, PBP2B_I452A/V, PBP2B_K479T, PBP2B_D512E, PBP2B_K513E, PBP2B_T515S, PBP2B_N606I, PBP2B_T625R, PBP2B_K674N | PBP2X_I72T, PBP2X_D136A, PBP2X_L204P, PBP2X_S450T, PBP2X_T551S, PBP2X_I568T, PBP2X_V593S, PBP2X_L597M, PBP2X_S604N, PBP2X_N616S, PBP2X_P622A, PBP2X_A627S/T, PBP2X_R628Q/Y/L, PBP2X_A630T/E, PBP2X_N631T/K/S, PBP2X_Q636K/R, PBP2X_A644S, PBP2X_Q655E/Y/N, PBP2X_A666S/T, PBP2X_K670N/Q, PBP2X_K678V/N/L/A, PBP2X_I680A, PBP2X_V685I, PBP2X_E694T/D, PBP2X_I701L |
| S186 | ST123 | 1 | 0,5 | 0,12 | PBP1A_T685A, PBP1A_A710S, PBP1A_G734D | PBP1B_E576K, PBP1B_T578A, PBP1B_A765P, PBP1B_S782A, PBP1B_N789T | PBP2A_H27R, PBP2A_I34V, PBP2A_I121V, PBP2A_N233D, PBP2A_A561S, PBP2A_S660T | PBP2B_P92S, PBP2B_D115N, PBP2B_K143E, PBP2B_N179S, PBP2B_S206A/V, PBP2B_I300V, PBP2B_R332K, PBP2B_N356E, PBP2B_S374A, PBP2B_S375G, PBP2B_T376S, PBP2B_I414V, PBP2B_Y432W, PBP2B_G433DEL, PBP2B_I452A/V, PBP2B_K479T, PBP2B_D512E, PBP2B_K513E, PBP2B_T515S, PBP2B_N606I, PBP2B_T625R, PBP2B_K674N | PBP2X_I72T, PBP2X_D136A, PBP2X_L204P, PBP2X_S450T, PBP2X_T551S, PBP2X_I568T, PBP2X_V593S, PBP2X_L597M, PBP2X_S604N, PBP2X_N616S, PBP2X_P622A, PBP2X_A627S/T, PBP2X_R628Q/Y/L, PBP2X_A630T/E, PBP2X_N631T/K/S, PBP2X_Q636K/R, PBP2X_A644S, PBP2X_Q655E/Y/N, PBP2X_A666S/T, PBP2X_K670N/Q, PBP2X_K678V/N/L/A, PBP2X_I680A, PBP2X_V685I, PBP2X_E694T/D, PBP2X_I701L |
| S306 | ST123 | 1 | 0,5 | 0,12 | PBP1A_T685A, PBP1A_A710S, PBP1A_G734D | PBP1B_E576K, PBP1B_T578A, PBP1B_A765P, PBP1B_S782A, PBP1B_N789T | PBP2A_H27R, PBP2A_I34V, PBP2A_I121V, PBP2A_N233D, PBP2A_A561S, PBP2A_S660T | PBP2B_P92S, PBP2B_D115N, PBP2B_K143E, PBP2B_N179S, PBP2B_S206A/V, PBP2B_I300V, PBP2B_R332K, PBP2B_N356E, PBP2B_S374A, PBP2B_S375G, PBP2B_T376S, PBP2B_I414V, PBP2B_Y432W, PBP2B_G433DEL, PBP2B_I452A/V, PBP2B_K479T, PBP2B_D512E, PBP2B_K513E, PBP2B_T515S, PBP2B_N606I, PBP2B_T625R, PBP2B_K674N | PBP2X_I72T, PBP2X_D136A, PBP2X_L204P, PBP2X_S450T, PBP2X_T551S, PBP2X_I568T, PBP2X_V593S, PBP2X_L597M, PBP2X_S604N, PBP2X_N616S, PBP2X_P622A, PBP2X_A627S/T, PBP2X_R628Q/Y/L, PBP2X_A630T/E, PBP2X_N631T/K/S, PBP2X_Q636K/R, PBP2X_A644S, PBP2X_Q655E/Y/N, PBP2X_A666S/T, PBP2X_K670N/Q, PBP2X_K678V/N/L/A, PBP2X_I680A, PBP2X_V685I, PBP2X_E694T/D, PBP2X_I701L |
| S309 | ST123 | 1 | 2 | 1 | PBP1A_T606P, PBP1A_T685A, PBP1A_A710S, PBP1A_G734D | PBP1B_E576K, PBP1B_T578A, PBP1B_A765P, PBP1B_S782A, PBP1B_N789T | PBP2A_H27R, PBP2A_I34V, PBP2A_I121V, PBP2A_N233D, PBP2A_A561S, PBP2A_S660T | PBP2B_P8S, 0, PBP2B_N63T, PBP2B_I66V, PBP2B_Q74K, PBP2B_K76T, PBP2B_E82Q, PBP2B_P92S, PBP2B_K143E, PBP2B_N179S, PBP2B_S206A/V, PBP2B_I300V, PBP2B_R332K, PBP2B_N356E, PBP2B_S374A, PBP2B_S375G, PBP2B_T376S, PBP2B_I414V, PBP2B_Y432W, PBP2B_G433DEL, PBP2B_I452A/V, PBP2B_K479T, PBP2B_T507I, PBP2B_D512E, PBP2B_K513E, PBP2B_T515S, PBP2B_D587E, PBP2B_T625R, PBP2B_K674N | PBP2X_P2S, PBP2X_K7R, PBP2X_K14R, PBP2X_K15R, PBP2X_S20T, PBP2X_R24Q, PBP2X_R25K, PBP2X_S29N, PBP2X_V37I, PBP2X_I43S, PBP2X_Y47L, PBP2X_F55A, PBP2X_D58S, PBP2X_R69Q/L, PBP2X_I72T, PBP2X_K76R, PBP2X_V97I, PBP2X_E104D, PBP2X_L109F, PBP2X_K111E, PBP2X_Q116E, PBP2X_S117A, PBP2X_S118T, PBP2X_F127L, PBP2X_K128N, PBP2X_H130Y, PBP2X_M139N, PBP2X_E140Q, PBP2X_A173T, PBP2X_E177K, PBP2X_L204P, PBP2X_L220M, PBP2X_I236L/V, PBP2X_V245I, PBP2X_K251N/T, PBP2X_T254Q/K, PBP2X_N279T, PBP2X_N284T, PBP2X_R288K, PBP2X_V295I, PBP2X_Q321A, PBP2X_L324M, PBP2X_L325I, PBP2X_V346L, PBP2X_Y361F, PBP2X_N364S, PBP2X_V367T, PBP2X_L378V, PBP2X_A380E/D, PBP2X_Y382K/M/Q, PBP2X_S383E, PBP2X_Y389F, PBP2X_M401V, PBP2X_Q405E, PBP2X_Q407E, PBP2X_F422Y, PBP2X_M437L, PBP2X_S445T, PBP2X_S450T, PBP2X_T467S, PBP2X_D486N, PBP2X_T491S, PBP2X_V494L/I, PBP2X_D511A, PBP2X_R514G, PBP2X_Y525F, PBP2X_D541E, PBP2X_V547M, PBP2X_T551S, PBP2X_I568T, PBP2X_N569K, PBP2X_N595S, PBP2X_R600N/D, PBP2X_D601T/S, PBP2X_A627S/T, PBP2X_K678V/N/L/A, PBP2X_K741E |
| S313 | ST123 | 2 | 1 | 1 | PBP1A_T606P, PBP1A_T685A, PBP1A_A710S, PBP1A_G734D | PBP1B_E576K, PBP1B_T578A, PBP1B_A765P, PBP1B_S782A, PBP1B_N789T | PBP2A_H27R, PBP2A_I34V, PBP2A_I121V, PBP2A_N233D, PBP2A_A561S, PBP2A_S660T | PBP2B_P8S, 0, PBP2B_N63T, PBP2B_I66V, PBP2B_Q74K, PBP2B_K76T, PBP2B_E82Q, PBP2B_P92S, PBP2B_K143E, PBP2B_N179S, PBP2B_S206A/V, PBP2B_I300V, PBP2B_R332K, PBP2B_N356E, PBP2B_S374A, PBP2B_S375G, PBP2B_T376S, PBP2B_I414V, PBP2B_Y432W, PBP2B_G433DEL, PBP2B_I452A/V, PBP2B_K479T, PBP2B_T507I, PBP2B_D512E, PBP2B_K513E, PBP2B_T515S, PBP2B_D587E, PBP2B_T625R, PBP2B_K674N | PBP2X_P2S, PBP2X_K7R, PBP2X_K14R, PBP2X_K15R, PBP2X_S20T, PBP2X_R24Q, PBP2X_R25K, PBP2X_S29N, PBP2X_V37I, PBP2X_I43S, PBP2X_Y47L, PBP2X_F55A, PBP2X_D58S, PBP2X_R69Q/L, PBP2X_I72T, PBP2X_K76R, PBP2X_V97I, PBP2X_E104D, PBP2X_L109F, PBP2X_K111E, PBP2X_Q116E, PBP2X_S117A, PBP2X_S118T, PBP2X_F127L, PBP2X_K128N, PBP2X_H130Y, PBP2X_M139N, PBP2X_E140Q, PBP2X_A173T, PBP2X_E177K, PBP2X_L204P, PBP2X_L220M, PBP2X_I236L/V, PBP2X_V245I, PBP2X_K251N/T, PBP2X_T254Q/K, PBP2X_N279T, PBP2X_N284T, PBP2X_R288K, PBP2X_V295I, PBP2X_Q321A, PBP2X_L324M, PBP2X_L325I, PBP2X_V346L, PBP2X_Y361F, PBP2X_N364S, PBP2X_V367T, PBP2X_L378V, PBP2X_A380E/D, PBP2X_Y382K/M/Q, PBP2X_S383E, PBP2X_Y389F, PBP2X_M401V, PBP2X_Q405E, PBP2X_Q407E, PBP2X_F422Y, PBP2X_M437L, PBP2X_S445T, PBP2X_S450T, PBP2X_T467S, PBP2X_D486N, PBP2X_T491S, PBP2X_V494L/I, PBP2X_D511A, PBP2X_R514G, PBP2X_Y525F, PBP2X_D541E, PBP2X_V547M, PBP2X_T551S, PBP2X_I568T, PBP2X_N569K, PBP2X_N595S, PBP2X_R600N/D, PBP2X_D601T/S, PBP2X_A627S/T, PBP2X_K678V/N/L/A, PBP2X_K741E |
| S365 | ST123 | 2 | 1 | 0,12 | PBP1A_T685A, PBP1A_A710S, PBP1A_G734D | PBP1B_E576K, PBP1B_T578A, PBP1B_A765P, PBP1B_S782A, PBP1B_N789T | PBP2A_H27R, PBP2A_I34V, PBP2A_I121V, PBP2A_N233D, PBP2A_A561S, PBP2A_S660T | PBP2B_P92S, PBP2B_D115N, PBP2B_K143E, PBP2B_N179S, PBP2B_S206A/V, PBP2B_I300V, PBP2B_R332K, PBP2B_N356E, PBP2B_S374A, PBP2B_S375G, PBP2B_T376S, PBP2B_I414V, PBP2B_Y432W, PBP2B_G433DEL, PBP2B_I452A/V, PBP2B_K479T, PBP2B_D512E, PBP2B_K513E, PBP2B_T515S, PBP2B_N606I, PBP2B_T625R, PBP2B_K674N | PBP2X_I72T, PBP2X_D136A, PBP2X_L204P, PBP2X_S450T, PBP2X_T551S, PBP2X_I568T, PBP2X_V593S, PBP2X_L597M, PBP2X_S604N, PBP2X_N616S, PBP2X_P622A, PBP2X_A627S/T, PBP2X_R628Q/Y/L, PBP2X_A630T/E, PBP2X_N631T/K/S, PBP2X_Q636K/R, PBP2X_A644S, PBP2X_Q655E/Y/N, PBP2X_A666S/T, PBP2X_K670N/Q, PBP2X_K678V/N/L/A, PBP2X_I680A, PBP2X_V685I, PBP2X_E694T/D, PBP2X_I701L |
| S412 | ST123 | 1 | 0,5 | 0,12 | PBP1A_T685A, PBP1A_A710S, PBP1A_G734D | PBP1B_E576K, PBP1B_T578A, PBP1B_A765P, PBP1B_S782A, PBP1B_N789T | PBP2A_H27R, PBP2A_I34V, PBP2A_I121V, PBP2A_N233D, PBP2A_A561S, PBP2A_S660T | PBP2B_P92S, PBP2B_D115N, PBP2B_K143E, PBP2B_N179S, PBP2B_S206A/V, PBP2B_I300V, PBP2B_R332K, PBP2B_N356E, PBP2B_S374A, PBP2B_S375G, PBP2B_T376S, PBP2B_I414V, PBP2B_Y432W, PBP2B_G433DEL, PBP2B_I452A/V, PBP2B_K479T, PBP2B_D512E, PBP2B_K513E, PBP2B_T515S, PBP2B_N606I, PBP2B_T625R, PBP2B_K674N | PBP2X_I72T, PBP2X_D136A, PBP2X_L204P, PBP2X_S450T, PBP2X_T551S, PBP2X_I568T, PBP2X_V593S, PBP2X_L597M, PBP2X_S604N, PBP2X_N616S, PBP2X_P622A, PBP2X_A627S/T, PBP2X_R628Q/Y/L, PBP2X_A630T/E, PBP2X_N631T/K/S, PBP2X_Q636K/R, PBP2X_A644S, PBP2X_Q655E/Y/N, PBP2X_A666S/T, PBP2X_K670N/Q, PBP2X_K678V/N/L/A, PBP2X_I680A, PBP2X_V685I, PBP2X_E694T/D, PBP2X_I701L |
| S425 | ST123 | 1 | 0,5 | 0,12 | PBP1A_T685A, PBP1A_A710S, PBP1A_G734D | PBP1B_E576K, PBP1B_T578A, PBP1B_A765P, PBP1B_S782A, PBP1B_N789T | PBP2A_H27R, PBP2A_I34V, PBP2A_I121V, PBP2A_N233D, PBP2A_A561S, PBP2A_S660T | PBP2B_P92S, PBP2B_D115N, PBP2B_K143E, PBP2B_N179S, PBP2B_S206A/V, PBP2B_I300V, PBP2B_R332K, PBP2B_N356E, PBP2B_S374A, PBP2B_S375G, PBP2B_T376S, PBP2B_I414V, PBP2B_Y432W, PBP2B_G433DEL, PBP2B_I452A/V, PBP2B_K479T, PBP2B_D512E, PBP2B_K513E, PBP2B_T515S, PBP2B_N606I, PBP2B_T625R, PBP2B_K674N | PBP2X_I72T, PBP2X_D136A, PBP2X_L204P, PBP2X_S450T, PBP2X_T551S, PBP2X_I568T, PBP2X_V593S, PBP2X_L597M, PBP2X_S604N, PBP2X_N616S, PBP2X_P622A, PBP2X_A627S/T, PBP2X_R628Q/Y/L, PBP2X_A630T/E, PBP2X_N631T/K/S, PBP2X_Q636K/R, PBP2X_A644S, PBP2X_Q655E/Y/N, PBP2X_A666S/T, PBP2X_K670N/Q, PBP2X_K678V/N/L/A, PBP2X_I680A, PBP2X_V685I, PBP2X_E694T/D, PBP2X_I701L |
| S426 | ST123 | 1 | 0,5 | 0,12 | PBP1A_T685A, PBP1A_A710S, PBP1A_G734D | PBP1B_E576K, PBP1B_T578A, PBP1B_A765P, PBP1B_S782A, PBP1B_N789T | PBP2A_H27R, PBP2A_I34V, PBP2A_I121V, PBP2A_N233D, PBP2A_A561S, PBP2A_S660T | PBP2B_P92S, PBP2B_D115N, PBP2B_K143E, PBP2B_N179S, PBP2B_S206A/V, PBP2B_I300V, PBP2B_R332K, PBP2B_N356E, PBP2B_S374A, PBP2B_S375G, PBP2B_T376S, PBP2B_I414V, PBP2B_Y432W, PBP2B_G433DEL, PBP2B_I452A/V, PBP2B_K479T, PBP2B_D512E, PBP2B_K513E, PBP2B_T515S, PBP2B_N606I, PBP2B_T625R, PBP2B_K674N | PBP2X_I72T, PBP2X_D136A, PBP2X_L204P, PBP2X_S450T, PBP2X_T551S, PBP2X_I568T, PBP2X_V593S, PBP2X_L597M, PBP2X_S604N, PBP2X_N616S, PBP2X_P622A, PBP2X_A627S/T, PBP2X_R628Q/Y/L, PBP2X_A630T/E, PBP2X_N631T/K/S, PBP2X_Q636K/R, PBP2X_A644S, PBP2X_Q655E/Y/N, PBP2X_A666S/T, PBP2X_K670N/Q, PBP2X_K678V/N/L/A, PBP2X_I680A, PBP2X_V685I, PBP2X_E694T/D, PBP2X_I701L |
| S427 | ST123 | 1 | 0,5 | 0,12 | PBP1A_T685A, PBP1A_A710S, PBP1A_G734D | PBP1B_E576K, PBP1B_T578A, PBP1B_A765P, PBP1B_S782A, PBP1B_N789T | PBP2A_H27R, PBP2A_I34V, PBP2A_I121V, PBP2A_N233D, PBP2A_A561S, PBP2A_S660T | PBP2B_P92S, PBP2B_D115N, PBP2B_K143E, PBP2B_N179S, PBP2B_S206A/V, PBP2B_I300V, PBP2B_R332K, PBP2B_N356E, PBP2B_S374A, PBP2B_S375G, PBP2B_T376S, PBP2B_I414V, PBP2B_Y432W, PBP2B_G433DEL, PBP2B_I452A/V, PBP2B_K479T, PBP2B_D512E, PBP2B_K513E, PBP2B_T515S, PBP2B_N606I, PBP2B_T625R, PBP2B_K674N | PBP2X_I72T, PBP2X_D136A, PBP2X_L204P, PBP2X_S450T, PBP2X_T551S, PBP2X_I568T, PBP2X_V593S, PBP2X_L597M, PBP2X_S604N, PBP2X_N616S, PBP2X_P622A, PBP2X_A627S/T, PBP2X_R628Q/Y/L, PBP2X_A630T/E, PBP2X_N631T/K/S, PBP2X_Q636K/R, PBP2X_A644S, PBP2X_Q655E/Y/N, PBP2X_A666S/T, PBP2X_K670N/Q, PBP2X_K678V/N/L/A, PBP2X_I680A, PBP2X_V685I, PBP2X_E694T/D, PBP2X_I701L |
| S435 | ST123 | 1 | 2 | 1 | PBP1A_T685A, PBP1A_A710S, PBP1A_G734D | PBP1B_E576K, PBP1B_T578A, PBP1B_A765P, PBP1B_S782A, PBP1B_N789T | PBP2A_H27R, PBP2A_I34V, PBP2A_I121V, PBP2A_N233D, PBP2A_A561S, PBP2A_S660T | PBP2B_P8S, PBP2B_N63T, PBP2B_I66V, PBP2B_Q74K, PBP2B_K76T, PBP2B_E82Q, PBP2B_P92S, PBP2B_K143E, PBP2B_N179S, PBP2B_S206A/V, PBP2B_I300V, PBP2B_R332K, PBP2B_N356E, PBP2B_S374A, PBP2B_S375G, PBP2B_T376S, PBP2B_I414V, PBP2B_Y432W, PBP2B_G433DEL, PBP2B_I452A/V, PBP2B_K479T, PBP2B_T507I, PBP2B_D512E, PBP2B_K513E, PBP2B_T515S, PBP2B_D587E, PBP2B_T625R, PBP2B_K674N | PBP2X_P2S, PBP2X_K7R, PBP2X_K14R, PBP2X_K15R, PBP2X_S20T, PBP2X_R24Q, PBP2X_R25K, PBP2X_S29N, PBP2X_V37I, PBP2X_I43S, PBP2X_Y47L, PBP2X_F55A, PBP2X_D58S, PBP2X_R69Q/L, PBP2X_I72T, PBP2X_K76R, PBP2X_V97I, PBP2X_E104D, PBP2X_L109F, PBP2X_K111E, PBP2X_Q116E, PBP2X_S117A, PBP2X_S118T, PBP2X_F127L, PBP2X_K128N, PBP2X_H130Y, PBP2X_M139N, PBP2X_E140Q, PBP2X_A173T, PBP2X_E177K, PBP2X_L204P, PBP2X_L220M, PBP2X_I236L/V, PBP2X_V245I, PBP2X_K251N/T, PBP2X_T254Q/K, PBP2X_N279T, PBP2X_N284T, PBP2X_R288K, PBP2X_V295I, PBP2X_Q321A, PBP2X_L324M, PBP2X_L325I, PBP2X_V346L, PBP2X_Y361F, PBP2X_N364S, PBP2X_V367T, PBP2X_L378V, PBP2X_A380E/D, PBP2X_Y382K/M/Q, PBP2X_S383E, PBP2X_Y389F, PBP2X_M401V, PBP2X_Q405E, PBP2X_Q407E, PBP2X_F422Y, PBP2X_M437L, PBP2X_S445T, PBP2X_S450T, PBP2X_T467S, PBP2X_D486N, PBP2X_T491S, PBP2X_V494L/I, PBP2X_D511A, PBP2X_R514G, PBP2X_Y525F, PBP2X_D541E, PBP2X_V547M, PBP2X_T551S, PBP2X_I568T, PBP2X_N569K, PBP2X_N595S, PBP2X_R600N/D, PBP2X_D601T/S, PBP2X_A627S/T, PBP2X_K678V/N/L/A, PBP2X_K741E |
| S436 | ST123 | 1 | 0,5 | 0,12 | PBP1A_T685A, PBP1A_A710S, PBP1A_G734D | PBP1B_E576K, PBP1B_T578A, PBP1B_A765P, PBP1B_S782A, PBP1B_N789T | PBP2A_H27R, PBP2A_I34V, PBP2A_I121V, PBP2A_N233D, PBP2A_A561S, PBP2A_S660T | PBP2B_P92S, PBP2B_D115N, PBP2B_K143E, PBP2B_N179S, PBP2B_S206A/V, PBP2B_I300V, PBP2B_R332K, PBP2B_N356E, PBP2B_S374A, PBP2B_S375G, PBP2B_T376S, PBP2B_I414V, PBP2B_Y432W, PBP2B_G433DEL, PBP2B_I452A/V, PBP2B_K479T, PBP2B_D512E, PBP2B_K513E, PBP2B_T515S, PBP2B_N606I, PBP2B_T625R, PBP2B_K674N | PBP2X_I72T, PBP2X_D136A, PBP2X_L204P, PBP2X_S450T, PBP2X_T551S, PBP2X_I568T, PBP2X_V593S, PBP2X_L597M, PBP2X_S604N, PBP2X_N616S, PBP2X_P622A, PBP2X_A627S/T, PBP2X_R628Q/Y/L, PBP2X_A630T/E, PBP2X_N631T/K/S, PBP2X_Q636K/R, PBP2X_A644S, PBP2X_Q655E/Y/N, PBP2X_A666S/T, PBP2X_K670N/Q, PBP2X_K678V/N/L/A, PBP2X_I680A, PBP2X_V685I, PBP2X_E694T/D, PBP2X_I701L |
| S437 | ST123 | 1 | 0,5 | 0,12 | PBP1A_T685A, PBP1A_A710S, PBP1A_G734D | PBP1B_E576K, PBP1B_T578A, PBP1B_A765P, PBP1B_S782A, PBP1B_N789T | PBP2A_H27R, PBP2A_I34V, PBP2A_I121V, PBP2A_N233D, PBP2A_A561S, PBP2A_S660T | PBP2B_P92S, PBP2B_D115N, PBP2B_K143E, PBP2B_N179S, PBP2B_S206A/V, PBP2B_I300V, PBP2B_R332K, PBP2B_N356E, PBP2B_S374A, PBP2B_S375G, PBP2B_T376S, PBP2B_I414V, PBP2B_Y432W, PBP2B_G433DEL, PBP2B_I452A/V, PBP2B_K479T, PBP2B_D512E, PBP2B_K513E, PBP2B_T515S, PBP2B_N606I, PBP2B_T625R, PBP2B_K674N | PBP2X_I72T, PBP2X_D136A, PBP2X_L204P, PBP2X_S450T, PBP2X_T551S, PBP2X_I568T, PBP2X_V593S, PBP2X_L597M, PBP2X_S604N, PBP2X_N616S, PBP2X_P622A, PBP2X_A627S/T, PBP2X_R628Q/Y/L, PBP2X_A630T/E, PBP2X_N631T/K/S, PBP2X_Q636K/R, PBP2X_A644S, PBP2X_Q655E/Y/N, PBP2X_A666S/T, PBP2X_K670N/Q, PBP2X_K678V/N/L/A, PBP2X_I680A, PBP2X_V685I, PBP2X_E694T/D, PBP2X_I701L |
| S438 | ST123 | 1 | 0,5 | 0,12 | PBP1A_T685A, PBP1A_A710S, PBP1A_G734D | PBP1B_E576K, PBP1B_T578A, PBP1B_A765P, PBP1B_S782A, PBP1B_N789T | PBP2A_H27R, PBP2A_I34V, PBP2A_I121V, PBP2A_N233D, PBP2A_A561S, PBP2A_S660T | PBP2B_P92S, PBP2B_D115N, PBP2B_K143E, PBP2B_N179S, PBP2B_S206A/V, PBP2B_I300V, PBP2B_R332K, PBP2B_N356E, PBP2B_S374A, PBP2B_S375G, PBP2B_T376S, PBP2B_I414V, PBP2B_Y432W, PBP2B_G433DEL, PBP2B_I452A/V, PBP2B_K479T, PBP2B_D512E, PBP2B_K513E, PBP2B_T515S, PBP2B_N606I, PBP2B_T625R, PBP2B_K674N | PBP2X_I72T, PBP2X_D136A, PBP2X_L204P, PBP2X_S450T, PBP2X_T551S, PBP2X_I568T, PBP2X_V593S, PBP2X_L597M, PBP2X_S604N, PBP2X_N616S, PBP2X_P622A, PBP2X_A627S/T, PBP2X_R628Q/Y/L, PBP2X_A630T/E, PBP2X_N631T/K/S, PBP2X_Q636K/R, PBP2X_A644S, PBP2X_Q655E/Y/N, PBP2X_A666S/T, PBP2X_K670N/Q, PBP2X_K678V/N/L/A, PBP2X_I680A, PBP2X_V685I, PBP2X_E694T/D, PBP2X_I701L |
| S443 | ST123 | 1 | 1 | 0,5 | PBP1A_S508N, PBP1A_T685A, PBP1A_A710S, PBP1A_G734D | PBP1B_E576K, PBP1B_T578A, PBP1B_A765P, PBP1B_S782A, PBP1B_N789T | PBP2A_H27R, PBP2A_I34V, PBP2A_I121V, PBP2A_N233D, PBP2A_A561S, PBP2A_S660T | PBP2B_P8S, PBP2B_N63T, PBP2B_I66V, PBP2B_Q74K, PBP2B_K76T, PBP2B_E82Q, PBP2B_P92S, PBP2B_K143E, PBP2B_N179S, PBP2B_S206A/V, PBP2B_I300V, PBP2B_R332K, PBP2B_N356E, PBP2B_S374A, PBP2B_S375G, PBP2B_T376S, PBP2B_I414V, PBP2B_Y432W, PBP2B_G433DEL, PBP2B_I452A/V, PBP2B_K479T, PBP2B_T507I, PBP2B_D512E, PBP2B_K513E, PBP2B_T515S, PBP2B_D587E, PBP2B_T625R, PBP2B_K674N | PBP2X_P2S, PBP2X_K7R, PBP2X_K14R, PBP2X_K15R, PBP2X_S20T, PBP2X_R24Q, PBP2X_R25K, PBP2X_S29N, PBP2X_V37I, PBP2X_I43S, PBP2X_Y47L, PBP2X_F55A, PBP2X_D58S, PBP2X_R69Q/L, PBP2X_I72T, PBP2X_K76R, PBP2X_V97I, PBP2X_E104D, PBP2X_L109F, PBP2X_K111E, PBP2X_Q116E, PBP2X_S117A, PBP2X_S118T, PBP2X_F127L, PBP2X_K128N, PBP2X_H130Y, PBP2X_M139N, PBP2X_E140Q, PBP2X_A173T, PBP2X_E177K, PBP2X_L204P, PBP2X_L220M, PBP2X_I236L/V, PBP2X_V245I, PBP2X_K251N/T, PBP2X_T254Q/K, PBP2X_N279T, PBP2X_N284T, PBP2X_R288K, PBP2X_V295I, PBP2X_Q321A, PBP2X_L324M, PBP2X_L325I, PBP2X_V346L, PBP2X_Y361F, PBP2X_N364S, PBP2X_V367T, PBP2X_L378V, PBP2X_A380E/D, PBP2X_Y382K/M/Q, PBP2X_S383E, PBP2X_Y389F, PBP2X_M401V, PBP2X_Q405E, PBP2X_Q407E, PBP2X_F422Y, PBP2X_M437L, PBP2X_S445T, PBP2X_S450T, PBP2X_T467S, PBP2X_D486N, PBP2X_T491S, PBP2X_V494L/I, PBP2X_D511A, PBP2X_R514G, PBP2X_Y525F, PBP2X_D541E, PBP2X_V547M, PBP2X_T551S, PBP2X_I568T, PBP2X_N569K, PBP2X_N595S, PBP2X_R600N/D, PBP2X_D601T/S, PBP2X_A627S/T, PBP2X_K678V/N/L/A, PBP2X_K741E |
| S475 | ST1953 | 1 | 2 | 0,5 | PBP1A_T685A, PBP1A_A710S, PBP1A_G734D | PBP1B_E576K, PBP1B_T578A, PBP1B_A765P, PBP1B_S782A, PBP1B_N789T | PBP2A_H27R, PBP2A_I34V, PBP2A_I121V, PBP2A_N233D, PBP2A_A561S, PBP2A_S660T | PBP2B_P8S, PBP2B_N63T, PBP2B_I66V, PBP2B_Q74K, PBP2B_K76T, PBP2B_E82Q, PBP2B_P92S, PBP2B_K143E, PBP2B_N179S, PBP2B_S206A/V, PBP2B_I300V, PBP2B_R332K, PBP2B_N356E, PBP2B_S374A, PBP2B_S375G, PBP2B_T376S, PBP2B_I414V, PBP2B_Y432W, PBP2B_G433DEL, PBP2B_I452A/V, PBP2B_K479T, PBP2B_T507I, PBP2B_D512E, PBP2B_K513E, PBP2B_T515S, PBP2B_D587E, PBP2B_T625R, PBP2B_K674N | PBP2X_P2S, PBP2X_K7R, PBP2X_K14R, PBP2X_K15R, PBP2X_S20T, PBP2X_R24Q, PBP2X_R25K, PBP2X_S29N, PBP2X_V37I, PBP2X_I43S, PBP2X_Y47L, PBP2X_F55A, PBP2X_D58S, PBP2X_R69Q/L, PBP2X_I72T, PBP2X_K76R, PBP2X_V97I, PBP2X_E104D, PBP2X_L109F, PBP2X_K111E, PBP2X_Q116E, PBP2X_S117A, PBP2X_S118T, PBP2X_F127L, PBP2X_K128N, PBP2X_H130Y, PBP2X_M139N, PBP2X_E140Q, PBP2X_A173T, PBP2X_E177K, PBP2X_L204P, PBP2X_L220M, PBP2X_I236L/V, PBP2X_V245I, PBP2X_K251N/T, PBP2X_T254Q/K, PBP2X_N279T, PBP2X_N284T, PBP2X_R288K, PBP2X_V295I, PBP2X_Q321A, PBP2X_L324M, PBP2X_L325I, PBP2X_V346L, PBP2X_Y361F, PBP2X_N364S, PBP2X_V367T, PBP2X_L378V, PBP2X_A380E/D, PBP2X_Y382K/M/Q, PBP2X_S383E, PBP2X_Y389F, PBP2X_M401V, PBP2X_Q405E, PBP2X_Q407E, PBP2X_F422Y, PBP2X_M437L, PBP2X_S445T, PBP2X_S450T, PBP2X_T467S, PBP2X_D486N, PBP2X_T491S, PBP2X_V494L/I, PBP2X_D511A, PBP2X_R514G, PBP2X_Y525F, PBP2X_D541E, PBP2X_V547M, PBP2X_T551S, PBP2X_I568T, PBP2X_N569K, PBP2X_N595S, PBP2X_R600N/D, PBP2X_D601T/S, PBP2X_A627S/T, PBP2X_K678V/N/L/A, PBP2X_K741E |
| S485 | ST1953 | 1 | 1 | 1 | PBP1A_T685A, PBP1A_A710S, PBP1A_G734D | PBP1B_E576K, PBP1B_T578A, PBP1B_A765P, PBP1B_S782A, PBP1B_N789T | PBP2A_H27R, PBP2A_I34V, PBP2A_I121V, PBP2A_N233D, PBP2A_A561S, PBP2A_S660T | PBP2B_P8S, PBP2B_N63T, PBP2B_I66V, PBP2B_Q74K, PBP2B_K76T, PBP2B_E82Q, PBP2B_P92S, PBP2B_K143E, PBP2B_N179S, PBP2B_S206A/V, PBP2B_I300V, PBP2B_R332K, PBP2B_N356E, PBP2B_S374A, PBP2B_S375G, PBP2B_T376S, PBP2B_I414V, PBP2B_Y432W, PBP2B_G433DEL, PBP2B_I452A/V, PBP2B_K479T, PBP2B_T507I, PBP2B_D512E, PBP2B_K513E, PBP2B_T515S, PBP2B_D587E, PBP2B_T625R, PBP2B_K674N | PBP2X_P2S, PBP2X_K7R, PBP2X_K14R, PBP2X_K15R, PBP2X_S20T, PBP2X_R24Q, PBP2X_R25K, PBP2X_S29N, PBP2X_V37I, PBP2X_I43S, PBP2X_Y47L, PBP2X_F55A, PBP2X_D58S, PBP2X_R69Q/L, PBP2X_I72T, PBP2X_K76R, PBP2X_V97I, PBP2X_E104D, PBP2X_L109F, PBP2X_K111E, PBP2X_Q116E, PBP2X_S117A, PBP2X_S118T, PBP2X_F127L, PBP2X_K128N, PBP2X_H130Y, PBP2X_M139N, PBP2X_E140Q, PBP2X_A173T, PBP2X_E177K, PBP2X_L204P, PBP2X_L220M, PBP2X_I236L/V, PBP2X_V245I, PBP2X_K251N/T, PBP2X_T254Q/K, PBP2X_N279T, PBP2X_N284T, PBP2X_R288K, PBP2X_V295I, PBP2X_Q321A, PBP2X_L324M, PBP2X_L325I, PBP2X_V346L, PBP2X_Y361F, PBP2X_N364S, PBP2X_V367T, PBP2X_L378V, PBP2X_A380E/D, PBP2X_Y382K/M/Q, PBP2X_S383E, PBP2X_Y389F, PBP2X_M401V, PBP2X_Q405E, PBP2X_Q407E, PBP2X_F422Y, PBP2X_M437L, PBP2X_S445T, PBP2X_S450T, PBP2X_T467S, PBP2X_D486N, PBP2X_T491S, PBP2X_V494L/I, PBP2X_D511A, PBP2X_R514G, PBP2X_Y525F, PBP2X_D541E, PBP2X_V547M, PBP2X_T551S, PBP2X_I568T, PBP2X_N569K, PBP2X_N595S, PBP2X_R600N/D, PBP2X_D601T/S, PBP2X_A627S/T, PBP2X_K678V/N/L/A, PBP2X_K741E |
| S494 | ST123 | 1 | 0,5 | 0,25 | PBP1A_T685A, PBP1A_A710S, PBP1A_G734D | PBP1B_E576K, PBP1B_T578A, PBP1B_A765P, PBP1B_S782A, PBP1B_N789T | PBP2A_H27R, PBP2A_I34V, PBP2A_I121V, PBP2A_N233D, PBP2A_A561S, PBP2A_S660T | PBP2B_P92S, PBP2B_D115N, PBP2B_K143E, PBP2B_N179S, PBP2B_S206A/V, PBP2B_I300V, PBP2B_R332K, PBP2B_N356E, PBP2B_S374A, PBP2B_S375G, PBP2B_T376S, PBP2B_I414V, PBP2B_Y432W, PBP2B_G433DEL, PBP2B_I452A/V, PBP2B_K479T, PBP2B_D512E, PBP2B_K513E, PBP2B_T515S, PBP2B_N606I, PBP2B_T625R, PBP2B_K674N | PBP2X_I72T, PBP2X_D136A, PBP2X_L204P, PBP2X_S450T, PBP2X_T551S, PBP2X_I568T, PBP2X_V593S, PBP2X_L597M, PBP2X_S604N, PBP2X_N616S, PBP2X_P622A, PBP2X_A627S/T, PBP2X_R628Q/Y/L, PBP2X_A630T/E, PBP2X_N631T/K/S, PBP2X_Q636K/R, PBP2X_A644S, PBP2X_Q655E/Y/N, PBP2X_A666S/T, PBP2X_K670N/Q, PBP2X_K678V/N/L/A, PBP2X_I680A, PBP2X_V685I, PBP2X_E694T/D, PBP2X_I701L |
| S511 | ST123 | 1 | 2 | 1 | PBP1A_T685A, PBP1A_A710S, PBP1A_G734D | PBP1B_E576K, PBP1B_T578A, PBP1B_A765P, PBP1B_S782A, PBP1B_N789T | PBP2A_H27R, PBP2A_I34V, PBP2A_I121V, PBP2A_N233D, PBP2A_A561S, PBP2A_S660T | PBP2B_P8S, PBP2B_N63T, PBP2B_I66V, PBP2B_Q74K, PBP2B_K76T, PBP2B_E82Q, PBP2B_P92S, PBP2B_K143E, PBP2B_N179S, PBP2B_S206A/V, PBP2B_I300V, PBP2B_R332K, PBP2B_N356E, PBP2B_S374A, PBP2B_S375G, PBP2B_T376S, PBP2B_I414V, PBP2B_Y432W, PBP2B_G433DEL, PBP2B_I452A/V, PBP2B_K479T, PBP2B_T507I, PBP2B_D512E, PBP2B_K513E, PBP2B_T515S, PBP2B_D587E, PBP2B_T625R, PBP2B_K674N | PBP2X_P2S, PBP2X_K7R, PBP2X_K14R, PBP2X_K15R, PBP2X_S20T, PBP2X_R24Q, PBP2X_R25K, PBP2X_S29N, PBP2X_V37I, PBP2X_I43S, PBP2X_Y47L, PBP2X_F55A, PBP2X_D58S, PBP2X_R69Q/L, PBP2X_I72T, PBP2X_K76R, PBP2X_V97I, PBP2X_E104D, PBP2X_L109F, PBP2X_K111E, PBP2X_Q116E, PBP2X_S117A, PBP2X_S118T, PBP2X_F127L, PBP2X_K128N, PBP2X_H130Y, PBP2X_M139N, PBP2X_E140Q, PBP2X_A173T, PBP2X_E177K, PBP2X_L204P, PBP2X_L220M, PBP2X_I236L/V, PBP2X_V245I, PBP2X_K251N/T, PBP2X_T254Q/K, PBP2X_N279T, PBP2X_N284T, PBP2X_R288K, PBP2X_V295I, PBP2X_Q321A, PBP2X_L324M, PBP2X_L325I, PBP2X_V346L, PBP2X_Y361F, PBP2X_N364S, PBP2X_V367T, PBP2X_L378V, PBP2X_A380E/D, PBP2X_Y382K/M/Q, PBP2X_S383E, PBP2X_Y389F, PBP2X_M401V, PBP2X_Q405E, PBP2X_Q407E, PBP2X_F422Y, PBP2X_M437L, PBP2X_S445T, PBP2X_S450T, PBP2X_T467S, PBP2X_D486N, PBP2X_T491S, PBP2X_V494L/I, PBP2X_D511A, PBP2X_R514G, PBP2X_Y525F, PBP2X_D541E, PBP2X_V547M, PBP2X_T551S, PBP2X_I568T, PBP2X_N569K, PBP2X_N595S, PBP2X_R600N/D, PBP2X_D601T/S, PBP2X_A627S/T, PBP2X_K678V/N/L/A, PBP2X_K741E |
| S533 | ST123 | 1 | 0,25 | 0,12 | PBP1A_T685A, PBP1A_A710S, PBP1A_G734D | PBP1B_E576K, PBP1B_T578A, PBP1B_A765P, PBP1B_S782A, PBP1B_N789T | PBP2A_H27R, PBP2A_I34V, PBP2A_I121V, PBP2A_N233D, PBP2A_A561S, PBP2A_S660T | PBP2B_P92S, PBP2B_D115N, PBP2B_K143E, PBP2B_N179S, PBP2B_S206A/V, PBP2B_I300V, PBP2B_R332K, PBP2B_N356E, PBP2B_S374A, PBP2B_S375G, PBP2B_T376S, PBP2B_I414V, PBP2B_Y432W, PBP2B_G433DEL, PBP2B_I452A/V, PBP2B_K479T, PBP2B_D512E, PBP2B_K513E, PBP2B_T515S, PBP2B_N606I, PBP2B_T625R, PBP2B_K674N | PBP2X_I72T, PBP2X_D136A, PBP2X_L204P, PBP2X_S450T, PBP2X_T551S, PBP2X_I568T, PBP2X_V593S, PBP2X_L597M, PBP2X_S604N, PBP2X_N616S, PBP2X_P622A, PBP2X_A627S/T, PBP2X_R628Q/Y/L, PBP2X_A630T/E, PBP2X_N631T/K/S, PBP2X_Q636K/R, PBP2X_A644S, PBP2X_Q655E/Y/N, PBP2X_A666S/T, PBP2X_K670N/Q, PBP2X_K678V/N/L/A, PBP2X_I680A, PBP2X_V685I, PBP2X_E694T/D, PBP2X_I701L |
| S575 | ST123 | 1 | 1 | 1 | PBP1A_T685A, PBP1A_A710S, PBP1A_G734D | PBP1B_E576K, PBP1B_T578A, PBP1B_A765P, PBP1B_S782A, PBP1B_N789T | PBP2A_H27R, PBP2A_I34V, PBP2A_I121V, PBP2A_N233D, PBP2A_A561S, PBP2A_S660T | PBP2B_P8S, PBP2B_N63T, PBP2B_I66V, PBP2B_Q74K, PBP2B_K76T, PBP2B_E82Q, PBP2B_P92S, PBP2B_K143E, PBP2B_N179S, PBP2B_S206A/V, PBP2B_I300V, PBP2B_R332K, PBP2B_N356E, PBP2B_S374A, PBP2B_S375G, PBP2B_T376S, PBP2B_I414V, PBP2B_Y432W, PBP2B_G433DEL, PBP2B_I452A/V, PBP2B_K479T, PBP2B_T507I, PBP2B_D512E, PBP2B_K513E, PBP2B_T515S, PBP2B_D587E, PBP2B_T625R, PBP2B_K674N | PBP2X_P2S, PBP2X_K7R, PBP2X_K14R, PBP2X_K15R, PBP2X_S20T, PBP2X_R24Q, PBP2X_R25K, PBP2X_S29N, PBP2X_V37I, PBP2X_I43S, PBP2X_Y47L, PBP2X_F55A, PBP2X_D58S, PBP2X_R69Q/L, PBP2X_I72T, PBP2X_K76R, PBP2X_V97I, PBP2X_E104D, PBP2X_L109F, PBP2X_K111E, PBP2X_Q116E, PBP2X_S117A, PBP2X_S118T, PBP2X_F127L, PBP2X_K128N, PBP2X_H130Y, PBP2X_M139N, PBP2X_E140Q, PBP2X_A173T, PBP2X_E177K, PBP2X_L204P, PBP2X_L220M, PBP2X_I236L/V, PBP2X_V245I, PBP2X_K251N/T, PBP2X_T254Q/K, PBP2X_N279T, PBP2X_N284T, PBP2X_R288K, PBP2X_V295I, PBP2X_Q321A, PBP2X_L324M, PBP2X_L325I, PBP2X_V346L, PBP2X_Y361F, PBP2X_N364S, PBP2X_V367T, PBP2X_L378V, PBP2X_A380E/D, PBP2X_Y382K/M/Q, PBP2X_S383E, PBP2X_Y389F, PBP2X_M401V, PBP2X_Q405E, PBP2X_Q407E, PBP2X_F422Y, PBP2X_M437L, PBP2X_S445T, PBP2X_S450T, PBP2X_T467S, PBP2X_D486N, PBP2X_T491S, PBP2X_V494L/I, PBP2X_D511A, PBP2X_R514G, PBP2X_Y525F, PBP2X_D541E, PBP2X_V547M, PBP2X_T551S, PBP2X_I568T, PBP2X_N569K, PBP2X_N595S, PBP2X_R600N/D, PBP2X_D601T/S, PBP2X_A627S/T, PBP2X_K678V/N/L/A, PBP2X_K741E |
| S590 | ST123 | 1 | 0,25 | 0,25 | PBP1A_T685A, PBP1A_A710S, PBP1A_G734D | PBP1B_E576K, PBP1B_T578A, PBP1B_A765P, PBP1B_S782A, PBP1B_N789T | PBP2A_H27R, PBP2A_I34V, PBP2A_I121V, PBP2A_N233D, PBP2A_A561S, PBP2A_S660T | PBP2B_P92S, PBP2B_D115N, PBP2B_K143E, PBP2B_N179S, PBP2B_S206A/V, PBP2B_I300V, PBP2B_R332K, PBP2B_N356E, PBP2B_S374A, PBP2B_S375G, PBP2B_T376S, PBP2B_I414V, PBP2B_Y432W, PBP2B_G433DEL, PBP2B_I452A/V, PBP2B_K479T, PBP2B_D512E, PBP2B_K513E, PBP2B_T515S, PBP2B_N606I, PBP2B_T625R, PBP2B_K674N | PBP2X_I72T, PBP2X_D136A, PBP2X_L204P, PBP2X_S450T, PBP2X_T551S, PBP2X_I568T, PBP2X_V593S, PBP2X_L597M, PBP2X_S604N, PBP2X_N616S, PBP2X_P622A, PBP2X_A627S/T, PBP2X_R628Q/Y/L, PBP2X_A630T/E, PBP2X_N631T/K/S, PBP2X_Q636K/R, PBP2X_A644S, PBP2X_Q655E/Y/N, PBP2X_A666S/T, PBP2X_K670N/Q, PBP2X_K678V/N/L/A, PBP2X_I680A, PBP2X_V685I, PBP2X_E694T/D, PBP2X_I701L |
| S593 | ST123 | 1 | 0,25 | 0,25 | PBP1A_T685A, PBP1A_A710S, PBP1A_G734D | PBP1B_E576K, PBP1B_T578A, PBP1B_A765P, PBP1B_S782A, PBP1B_N789T | PBP2A_H27R, PBP2A_I34V, PBP2A_I121V, PBP2A_N233D, PBP2A_A561S, PBP2A_S660T | PBP2B_P92S, PBP2B_D115N, PBP2B_K143E, PBP2B_N179S, PBP2B_S206A/V, PBP2B_I300V, PBP2B_R332K, PBP2B_N356E, PBP2B_S374A, PBP2B_S375G, PBP2B_T376S, PBP2B_I414V, PBP2B_Y432W, PBP2B_G433DEL, PBP2B_I452A/V, PBP2B_K479T, PBP2B_D512E, PBP2B_K513E, PBP2B_T515S, PBP2B_N606I, PBP2B_T625R, PBP2B_K674N | PBP2X_I72T, PBP2X_D136A, PBP2X_L204P, PBP2X_S450T, PBP2X_T551S, PBP2X_I568T, PBP2X_V593S, PBP2X_L597M, PBP2X_S604N, PBP2X_N616S, PBP2X_P622A, PBP2X_A627S/T, PBP2X_R628Q/Y/L, PBP2X_A630T/E, PBP2X_N631T/K/S, PBP2X_Q636K/R, PBP2X_A644S, PBP2X_Q655E/Y/N, PBP2X_A666S/T, PBP2X_K670N/Q, PBP2X_K678V/N/L/A, PBP2X_I680A, PBP2X_V685I, PBP2X_E694T/D, PBP2X_I701L |
| S602 | ST94 | 1 | 1 | 0,12 | PBP1A_T685A, PBP1A_A710S, PBP1A_G734D | PBP1B_E576K, PBP1B_T578A, PBP1B_A765P, PBP1B_S782A, PBP1B_N789T | PBP2A_H27R, PBP2A_I34V, PBP2A_I121V, PBP2A_N233D, PBP2A_A561S, PBP2A_S660T | PBP2B_R19C, PBP2B_N179S, PBP2B_S206A/V, PBP2B_P234L, PBP2B_Q291E, PBP2B_I300V, PBP2B_E304K, PBP2B_R332K, PBP2B_A340S, PBP2B_V408I, PBP2B_I414V, PBP2B_Q415L, PBP2B_Y432W, PBP2B_G433DEL, PBP2B_I452A/V, PBP2B_K479T, PBP2B_T507I, PBP2B_D512E, PBP2B_K513E, PBP2B_T515S, PBP2B_D587E, PBP2B_T625R, PBP2B_K674N | PBP2X_I72T, PBP2X_L204P, PBP2X_Y382K/M/Q, PBP2X_Y389F, PBP2X_Q407E, PBP2X_T418A, PBP2X_F422Y, PBP2X_M437L, PBP2X_S445T, PBP2X_S450T, PBP2X_A460V, PBP2X_T467S, PBP2X_D486N, PBP2X_T491S, PBP2X_V494L/I, PBP2X_D511A, PBP2X_R514G, PBP2X_Y525F, PBP2X_D541E, PBP2X_V547M, PBP2X_T551S, PBP2X_Q560L, PBP2X_I568T, PBP2X_N595S, PBP2X_R600N/D, PBP2X_D601T/S, PBP2X_S621A/V, PBP2X_A625S, PBP2X_A627S/T, PBP2X_R628Q/Y/L, PBP2X_N631T/K/S, PBP2X_Q636K/R, PBP2X_L640I, PBP2X_I641T/V, PBP2X_S648T, PBP2X_Q655E/Y/N, PBP2X_Q658H, PBP2X_V660I, PBP2X_A666S/T, PBP2X_K670N/Q, PBP2X_V671A, PBP2X_S677T, PBP2X_K678V/N/L/A, PBP2X_I680A, PBP2X_D707G, PBP2X_F709I, PBP2X_K720E, PBP2X_T722S/Q, PBP2X_S724T |

Table S4 - a. Effect of the PBP-patterns on the MIC value to penicillin log transformed.

| Outcome variable: MIC value to penicillin log transformed | | Adj. R-squared=0.685 | |
| --- | --- | --- | --- |
| Factor | Coefficient | 95%CI | *p* |
| PBP-pattern 4 | 0.280 | [ 0.131 ; 0.428] | <0.001 |
| PBP-pattern 5 | 0.529 | [ 0.382 ; 0.676] | <0.001 |

Table S4 - b. Effect of the PBP-patterns on the MIC value to ceftiofur log transformed.

| Outcome variable: MIC value to ceftiofur log transformed | | Adj. R-squared=0.650 | |
| --- | --- | --- | --- |
| Factor | Coefficient | 95%CI | *p* |
| PBP-pattern 4 | 0.200 | [ 0.105 ; 0.295] | <0.001 |
| PBP-pattern 9 | 0.455 | [ 0.360 ; 0.550] | <0.001 |
